# High expression of metabolic enzyme PFKFB4 is associated with poor prognosis of operable breast cancer

**DOI:** 10.1186/s12935-019-0882-2

**Published:** 2019-06-18

**Authors:** Ling Yao, Lei Wang, Zhi-Gang Cao, Xin Hu, Zhi-Ming Shao

**Affiliations:** 1 Department of Breast Surgery, Key Laboratory of Breast Cancer in Shanghai, Fudan University Shanghai Cancer Center, Fudan University, 270 Dong-An Rd., Shanghai, 200032 People’s Republic of China; 2 Key Laboratory of Breast Cancer in Shanghai, Fudan University Shanghai Cancer Center, Fudan University, Shanghai, People’s Republic of China; 3 Department of Oncology, Shanghai Medical College, Fudan University, Shanghai, People’s Republic of China; 4 Institutes of Biomedical Sciences, Fudan University, Shanghai, People’s Republic of China

**Keywords:** PFKFB4, Overall survival, Disease-free survival, Breast cancer

## Abstract

**Background:**

Enhanced glycolysis in tumors, known as the Warburg effect, provides the metabolic basis of enhanced cancer cell proliferation and metastasis. The Warburg pathway enzyme 6-phosphofructo-2-kinase/fructose-2,6-bisphosphatase 4 (PFKFB4) is a newly identified key kinase that regulates transcriptional reprogramming and cell proliferation. Here we show the prognostic value of PFKFB4 expression in patients with operable breast cancer.

**Methods:**

PFKFB4 expression was evaluated by immunohistochemistry in surgical specimens retrospectively collected from 200 patients with histologically proven invasive ductal breast cancer. Kaplan–Meier survival analysis and Cox regression analysis were performed to assess the prognostic significance of PFKFB4 expression.

**Results:**

Kaplan–Meier survival analysis revealed that breast cancer patients with high PFKFB4 expression demonstrated unfavorable disease-free survival (*p *= 0.008) and overall survival (*p *= 0.002). PFKFB4 had an hazard ratio (HR) of 7.38 (95% CI 1.69–32.3; *p* = 0.008) in univariate Cox analysis and retained prognostic power (HR 7.44, 95% CI 1.67–33.2; *p* = 0.009) when adjusted by tumor size, lymph node status, grade, estrogen receptor status, human epidermal growth factor receptor 2 status and subtype, which indicated PFKFB4 was an independent prognostic factor in breast cancer.

**Conclusions:**

Together, our findings establish the prognostic value of metabolic enzyme PFKFB4 in patients with operable breast cancer.

**Electronic supplementary material:**

The online version of this article (10.1186/s12935-019-0882-2) contains supplementary material, which is available to authorized users.

## Background

Breast cancer is the most common cancer in women cancer and the second highest cause of cancer-related death in women in the United States [1]. In China, breast cancer is also the most commonly diagnosed cancer and accounts for 15% of all new cancers in women [2]. Breast cancer is a heterogeneous disease composed of four subtypes: luminal A, luminal B, human epidermal growth factor receptor 2 (HER-2)-enriched and triple-negative [3]. According to current guidelines and consensus, the selection of an appropriate adjuvant therapy regimen is largely based on the subtype and risk recurrence category. The value of prognostic biomarkers and gene-based assays has recently been added into the 8th edition of the primary tumor, lymph node, and metastasis (TNM) classification of the American Joint Commission of Cancer for breast cancer [4]. Despite the intensive research into the mechanisms of breast cancer performed over the past decades, very few of the identified critical molecules have been adopted for therapeutic or prognostic approaches in clinical practice. Thus, there is an urgent need to identify novel biomarkers that provide additional risk assessment for personalized treatment.

In recent years, studies of cancer metabolism have provided insight into the process of adaptation of cancer cells, which alter their metabolic activity to meet the increased needs for energy and biosynthetic precursors. Enhanced glycolysis, known as the Warburg effect, is observed in many cancers and provides the metabolic basis of cancer cell proliferation [5]. The Warburg pathway enzyme 6-phosphofructo-2-kinase/fructose-2,6-bisphosphatase 4 (PFKFB4) is a newly identified key factor that regulate transcriptional reprogramming and functions as the most dominant kinase that regulates cell proliferation [6]. Several studies identified PFKFB4 as a key molecule in multiple cancers, including breast cancer [6], prostate cancer [7] and glioma [8]. However, our current knowledge about PFKFB4 has largely originated from in vitro studies, and the in vivo relevance of this molecule requires further study. Furthermore, the prognostic value of the PFKFB4 protein in breast cancer has not been investigated.

In this study, we evaluated the prognostic power of PFKFB4 expression in 200 tumor samples from patients with stage I to III breast cancer. We examined the associations between PFKFB4, clinicopathological variables and survival. Our results indicated that elevated PFKFB4 is strongly associated with shorter disease-free survival (DFS) and overall survival (OS) and indicate that PFKFB4 could serve as a novel prognostic factor in breast cancer.

## Materials and methods

### Patients and specimens

Clinical data and surgical specimens were retrospectively collected from 200 female patients who were diagnosed with stage I to III primary breast cancer at the Department of Breast Surgery in Fudan University Shanghai Cancer Center (FUSCC, Shanghai, China) between January 2004 and January 2008. All specimens in this cohort were histologically confirmed with invasive ductal carcinoma and all participants underwent a mastectomy and axillary lymph node dissection or breast conservation surgery. Treatment decisions, including chemotherapy, radiation therapy, target therapy or endocrine therapy, were performed at the discretion of clinicians following the Chinese Anti-Cancer Association’s guidelines. All patients were regularly followed, with the last update occurring in October 2014. This study was approved by the Ethics Committee of FUSCC, and written informed consent was acquired from all patients.

Data of the clinicopathological variables and expression status of estrogen receptor (ER), progesterone receptor (PR) and human epidermal growth factor receptor 2 (HER-2) in the surgical specimens were retrospectively collected from the medical database of FUSCC. Since Ki-67 expression status was not available in all cases, we defined breast cancer subtypes as follows: luminal (ER and/or PR positive, HER-2 negative); HER-2-enriched (ER and PR negative, HER-2 positive); and triple-negative (ER negative, PR negative, HER-2 negative).

### Tissue microarray preparation

Tissue microarrays (TMAs) were constructed as previously described [9]. Briefly, breast cancer specimens from the 200 surgical cases described above were fixed using standard protocols. Archived and de-identified formalin-fixed paraffin-embedded (FFPE) samples were then analyzed. After histological examination of the tissue samples by our dedicated pathologist, TMAs were developed by punching two 10-mm-diameter cores out of each tumor at two different sites. The TMAs were prepared using a Quick-Ray tissue arrayer (Unitma Co., Ltd., Seoul, South Korea) at the Department of Pathology in FUSCC. The use of the tissue samples was approved by the Institutional Review Board at FUSCC.

### Immunohistochemistry staining and evaluation

As in our previous study [9, 10], the TMAs were subjected to immunohistochemical staining for PFKFB4 using a two-step protocol (GTVision™ III). PFKFB4 was detected using the rabbit anti-PFKFB4 polyclonal antibody [ab137785] (1:50;Abcam). The negative controls were generated using phosphate-buffered saline instead of primary antibody. Positive controls were established according to the instructions provided with the antibodies. The PFKFB4 staining intensity was rated according to four scores (0 denoting negative; 1, weak; 2, moderate; and 3, strong). The stained TMAs were evaluated independently by two experienced pathologists who were blinded to all clinical data on a case-by-case basis. Because the TMAs represented duplicate samples for each case, the score used in all subsequent analyses was the average across the available scores. The cutoff value for high and low PFKFB4 expression was the median value of the intensity scores.

### Statistical analysis

Patient clinicopathological variables were summarized for all participants using standard descriptive statistics. The Pearson χ^2^ test was performed to compare qualitative variables, and Fisher’s exact test was used when necessary. DFS was defined as the time from the date of primary surgery to the date of recurrence, distant metastasis or death. OS was calculated from the date of primary surgery to the date of death. The follow-up period was defined as the time from surgery to relapse or death (for complete observations) or to the last observation (for censored cases).

Survival analysis was performed using the Kaplan–Meier method and log-rank test was used to test the differences in survival by covariates. Reverse Kaplan–Meier method was used to calculate the median follow-up time. Univariate Cox regression models were fitted to estimate the effect of clinicopathological variables and PFKFB4 at the time-to-event endpoints (DFS and OS events). Multivariate analyses were performed to estimate the risk of variables in which Wald *p* were smaller than 0.20 in univariate analyses. All statistical analyses were performed using SPSS statistics version 24 (SPSS Inc., Chicago, IL, USA). All reported *p* values were two-sided, and *p* < 0.05 was considered statistically significant.

## Results

### Expression pattern of PFKFB4 in breast cancer

**Fig. 1:**
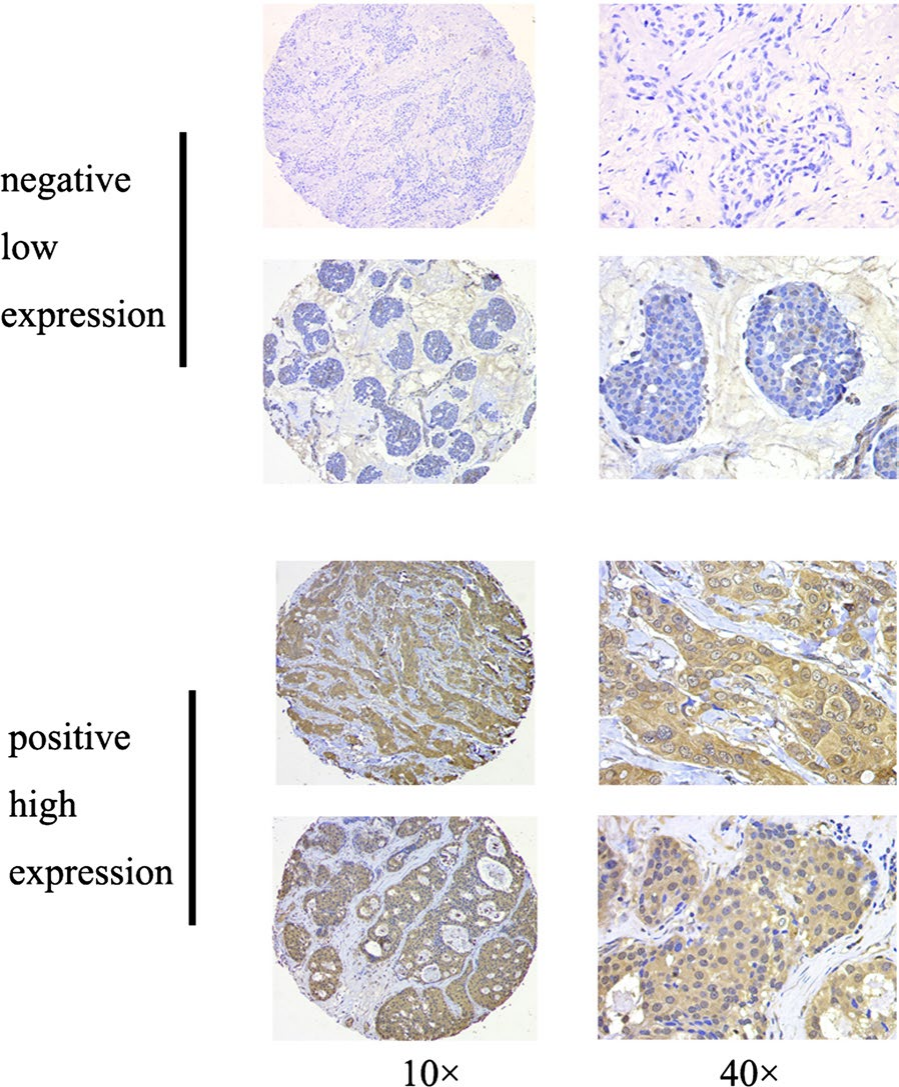
Representative PFKFB4 immunohistochemical staining in breast cancer specimens in low-magnification (×100) and high-magnification (×400) images. The PFKFB4 staining intensities were classified as negative (low expression) and positive (high expression)

A total of 200 female patients with invasive ductal breast cancers were recruited in this study. The average age of the participants was 50.4 years (standard deviation 10.4, median 50.0, range 26–84). The clinicopathological characteristics of the cohort are summarized in Table 1. After a median follow-up time of 93.9 months, 37 of the 200 patients experienced recurrence or death. To investigate the clinical significance of PFKFB4 in breast cancer, we conducted immunohistochemical staining for PFKFB4 expression in the tumor samples. As shown in Fig. 1, positive staining for PFKFB4 protein was observed mainly in the cytoplasm of breast cancer cells, and most of the intra- or extra-tumor stromal cells were negative for PFKFB4. Using the semi-quantified scoring criteria, positive PFKFB4 staining was observed in 98 (49.0%) of the 200 cases (Table 1). Higher PFKFB4 expression was associated with ER status (*p* = 0.021) and had a borderline relationship with PR status (*p* = 0.059) and subtype (*p* = 0.068).

**Table 1:** Clinicaopathological variables and the expression of PFKFB4

Variable	Number of patients	PFKFB4 expression		*p*-value^a^
		Negative number (%)	Positive number (%)	
Total	200	102 (51.0)	98 (49.0)	
Age (years)				0.453
≤ 50	113	55 (48.7)	58 (51.3)	
> 50	87	47 (54.0)	40 (46.0)	
Menopausal status				0.837
Premenopausal	121	61 (50.4)	60 (49.6)	
Postmenopausal	79	41 (51.9)	38 (48.1)	
Tumor size (cm)				0.168
≤ 2	95	50 (52.6)	45 (47.4)	
> 2, ≤ 5	91	48 (52.7)	43 (47.3)	
> 5	8	1 (12.5)	7 (87.5)	
Cannot be measured	6	3 (50.0)	3 (50.0)	
Lymph node status				0.890
Negative	99	50 (50.5)	49 (49.5)	
Positive	101	52 (51.5)	49 (48.5)	
Grade				0.716
Grade 1	2	1 (50.0)	1 (50.0)	
Grade 2	94	52 (55.3)	42 (44.7)	
Grade 3	56	26 (46.4)	30 (53.6)	
Unknown	48	23 (47.9)	25 (52.1)	
ER status				0.021
Negative	112	49 (43.8)	63 (56.3)	
Positive	88	53 (60.2)	35 (39.8)	
PR status				0.059
Negative	113	51 (45.1)	62 (54.9)	
Positive	87	51 (58.6)	36 (41.4)	
HER2 status				0.120
Negative	99	45 (45.5)	54 (54.5)	
Positive	101	57 (56.4)	44 (43.6)	
Subtype^b^				0.068
Luminal	100	58 (58.0)	42 (42.0)	
HER2-enriched	50	25 (50.0)	25 (50.0)	
Triple-negative	50	19 (38.0)	31 (62.0)	

*ER* estrogen receptor, *HER-2* human epidermal growth factor receptor 2, *PFKFB4* 6-phosphofructo-2-kinase/fructose-2,6-bisphosphatase 4, *PR* progesterone receptor

^a^Based on the Pearson χ^2^ test (Fisher’s exact test was used when needed)

^b^Definitions of subtypes: luminal (ER- and/or PR-positive), HER-2-enriched (ER- and PR-negative, HER-2-positive), and triple-negative (ER-negative, PR-negative, and HER-2-negative)

### Elevated PFKFB4 expression is associated with poor DFS and OS in breast cancer

**Fig. 2:**
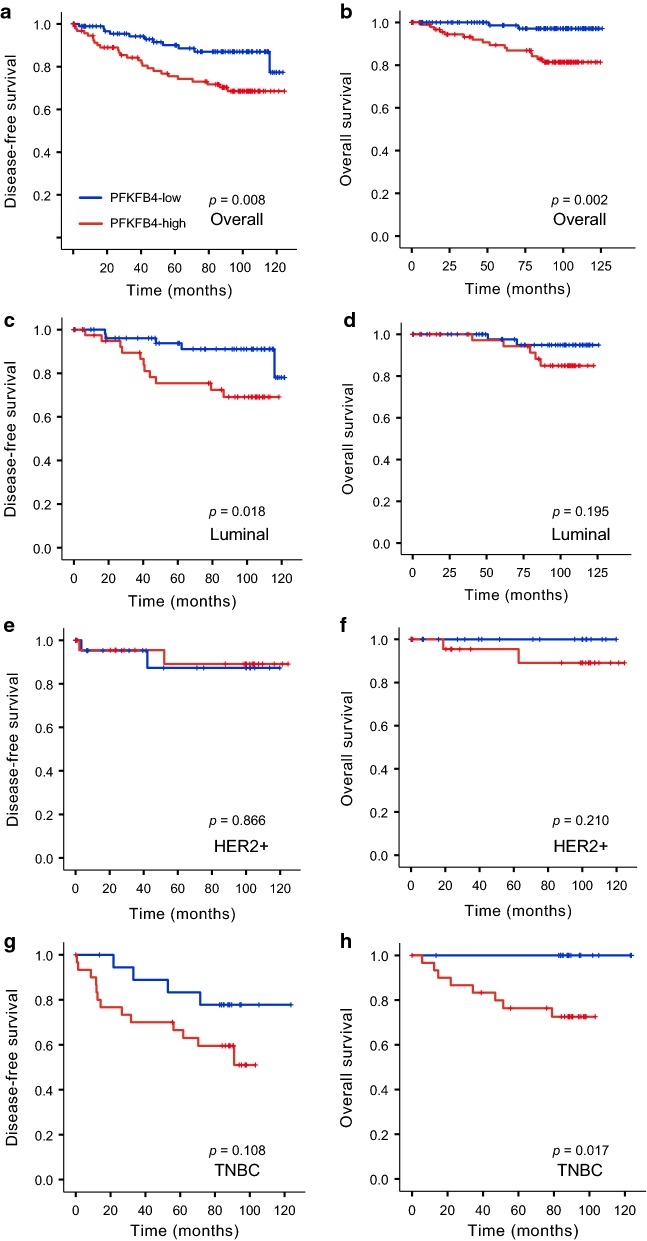
Kaplan–Meier analysis of PFKFB4 in breast cancer. **a** DFS in all patients (*n* = 200); **b** OS (*n* = 200); **c** DFS in luminal subgroup (*n* = 100); **d** OS in luminal subgroup (*n* = 100); **e** DFS in HER-2-enriched subgroup (*n* = 50); **f** OS in HER-2 in HER-2-enriched subgroup (*n* = 50); **g** DFS in triple-negative subgroup (*n* = 50); **h** OS in triple-negative subgroup (*n* = 50). DFS, disease-free survival; OS, overall survival; HER-2, human epidermal growth factor receptor 2. Definitions of subtypes: luminal (ER- and/or PR-positive), HER-2-enriched (ER- and PR-negative, HER-2-positive), and triple-negative (ER-negative, PR-negative, and HER-2-negative)

To evaluate the clinical implications of PFKFB4 overexpression in breast cancer, we assessed the correlation between PFKFB4 status and DFS and OS using Kaplan–Meier survival analysis. As shown in Fig. 2a, b, patients with high PFKFB4 expression showed unfavorable DFS (*p* = 0.008) and OS (*p *= 0.002). After stratification by molecular subtypes, PFKFB4 predicted poor DFS in luminal breast cancer (*p* = 0.018; Fig. 2c) and better OS was observed in TNBC patients with low PFKFB4 expression (*p* = 0.017; Fig. 2h). However, no survival association was observed in the HER-2-enriched subgroup (*p* > 0.05; Fig. 2e, f). There was no positive correlation between PFKFB4 and OS in the luminal subtype (*p* > 0.05; Fig. 2d) and OS showed no significance in TNBC patients when stratified by PFKFB2 status (*p* > 0.05; Fig. 2g). These results suggest that the molecular function of PFKFB4 in breast cancer may depend on the molecular subtype, which is largely determined by receptor status.

Moreover, since PFKFB4 can regulate ER [6], we further investigated the survival relationship of PFKFB4 in breast cancer patients stratified by ER status. High PFKFB4 was associated with poor DFS in ER-positive patients (*p* = 0.020; Additional file 1: Figure S1A), whereas low expression of PFKFB4 predicted favorable long-term outcome in ER-negative patients (*p* = 0.004; Additional file 1: Figure S1D). No positive correlation between PFKFB4 expression and OS was observed (*p* > 0.05; Additional file 1: Figure S1B), and comparison of DFS in ER-negative subgroups revealed no significant difference when stratified by PFKFB4 status (*p* > 0.05; Additional file 1: Figure S1C).

### Univariate and multivariate Cox regression analyses

Next, we examined the correlation between DFS and the clinicopathological variables in the univariate Cox analyses. As shown in Table 2, PFKFB4 (HR 2.52, 95% CI 1.24–5.11; *p* = 0.010) and triple-negative subtype (HR 2.19, 95% CI 1.10–4.35; *p* = 0.025) were associated with a higher risk of relapse and death. After adjustment by variables for which HR were smaller than 0.2, PFKFB4 remained a significant prognostic factor with an HR of 2.42 (95% CI 1.17–5.00; *p* = 0.017).

**Table 2:** Univariate and multivariate analysis of clinicopathological variables and PFKFB4 associated with disease-free survival

Variable	Univariate		Multivariate	
	HR (95% CI)	*p* value	HR (95% CI)	*p*-value
Age (years)				
≤ 50	1	–	–	–
> 50	1.12 (0.58–2.14)	0.734	–	–
Menopausal status				
Premenopausal	1	–	–	–
Postmenopausal	1.13 (0.58–2.17)	0.726	–	–
Tumor size (cm)				
≤ 2	1	–	–	–
> 2, ≤ 5	1.09 (0.56–2.12)	0.800	–	–
> 5	1.00 (0.13–7.49)	0.998	–	–
Cannot be measured	1.29 (0.17–9.69)	0.807	–	–
Lymph node status				
Negative	1	–	–	–
Positive	1.46 (0.76–2.81)	0.253	–	–
Grade				
Grade 1–2	1	–	–	–
Grade 3	1.10 (0.50–2.43)	0.810	–	–
Unknown	1.53 (0.71–3.29)	0.281	–	–
ER status				
Negative	1	–	1	–
Positive	0.58 (0.29–1.14)	0.112	0.51 (0.13–2.09)	0.353
PR status				
Negative	1	–	–	–
Positive	0.66 (0.34–1.30)	0.230	–	–
HER2 status				
Negative	1	–	1	–
Positive	0.62 (0.32–1.22)	0.166	1.26 (0.44–3.61)	0.671
Subtype^a^				
Luminal	1	–	1	–
HER2-enriched	0.61 (0.20–1.82)	0.373	0.26 (0.059–1.19)	0.083
Triple-negative	2.19 (1.10–4.35)	0.025	1.22 (0.24–6.06)	0.812
PFKFB4				
Negative	1	–	1	–
Positive	2.52 (1.24–5.11)	0.010	2.42 (1.17–5.00)	0.017

*−* no data, *CI* confidence interval, *ER* estrogen receptor, *HER-2* human epidermal growth factor receptor 2, *HR* hazard ratio, *PFKFB4* 6-phosphofructo-2-kinase/fructose-2,6-bisphosphatase 4, *PR* progesterone receptor

^a^Definitions of subtypes: luminal (ER- and/or PR-positive), HER-2-enriched (ER- and PR-negative, HER-2-positive), and triple-negative (ER-negative, PR-negative, and HER-2-negative)

Table 3 presents the association between OS and the clinicopathological variables analyzed using univariate and multivariate Cox regression. PFKFB4 had an HR of 7.38 (95% CI 1.69–32.3; *p* = 0.008) and retained prognostic power (HR 7.44, 95% CI 1.67–33.2; *p* = 0.009) when adjusted by tumor size, lymph node status, grade, ER status, HER2 status and subtypes. Lymph node status, a traditional prognostic factor, was associated with poor OS as well (HR 3.41, 95% CI 1.08–10.8; *p* = 0.037). Thus, PFKFB4 was established to be an independent prognostic factor in breast cancer.

**Table 3:** Univariate and multivariate analysis of clinicopathological variables and PFKFB4 associated with overall survival

Variable	Univariate		Multivariate	
	HR (95% CI)	*p*-value	HR (95% CI)	*p*-value
Age (years)				
≤ 50	1	–	–	–
> 50	1.72 (0.66–4.46)	0.266	–	–
Menopausal status				
Premenopausal	1	–	–	–
Postmenopausal	1.49 (0.58–3.87)	0.411	–	–
Tumor size (cm)				
≤ 2	1	–	1	–
> 2, ≤ 5	1.68 (0.60–4.71)	0.327	2.07 (0.69–6.18)	0.193
> 5	3.22 (0.39–26.8)	0.279	3.43 (0.33–36.0)	0.304
Cannot be measured	4.21 (0.50–35.1)	0.184	2.67 (0.24–29.7)	0.425
Lymph node status				
Negative	1	–	1	–
Positive	2.64 (0.93–7.49)	0.069	3.41 (1.08–10.8)	0.037
Grade				
Grade 1–2	1	–	1	–
Grade 3	2.36 (0.75–7.45)	0.142	2.01 (0.59–6.85)	0.264
Unknown	2.22 (0.64–7.65)	0.209	2.56 (0.64–10.2)	0.183
ER status				
Negative	1	–	–	–
Positive	0.64 (0.24–1.72)	0.371	–	–
PR status				
Negative	1	–	–	–
Positive	0.70 (0.26–1.89)	0.477	–	–
HER2 status				
Negative	1	–	1	–
Positive	0.49 (0.17–1.40)	0.184	0.67 (0.14–3.16)	0.617
Subtype^a^				
Luminal	1	–	1	–
HER2-enriched	0.71 (0.15–3.44)	0.675	0.47 (0.068–3.21)	0.439
Triple-negative	2.10 (0.76–5.79)	0.152	1.13 (0.31–4.17)	0.850
PFKFB4				
Negative	1	–	1	–
Positive	7.38 (1.69–32.3)	0.008	7.44 (1.67–33.2)	0.009

*−* no data, *CI* confidence interval, *ER* estrogen receptor, *HER-2* human epidermal growth factor receptor 2, *HR* hazard ratio, *PFKFB4* 6-phosphofructo-2-kinase/fructose-2,6-bisphosphatase 4, *PR* progesterone receptor

^a^Definitions of subtypes: luminal (ER- and/or PR-positive), HER-2-enriched (ER- and PR-negative, HER-2-positive), and triple-negative (ER-negative, PR-negative, and HER-2-negative)

## Discussion

Increasing recognition of the active role of cancer metabolism in tumorigenesis has led to the identification of novel markers for prognostic prediction [11, 12]. Enzymes participating in core metabolic pathways have proven to be essential for the proliferation and survival of cancer cells [6, 7, 13, 14]. In this study, we evaluated the relationship of the cancer metabolic enzyme PFKFB4 with the risk of recurrence, metastasis and death in operable breast cancer. We demonstrated that elevated PFKFB4 expression from immunohistochemistry analysis is associated with shorter DFS and OS in breast cancer. Our results established that PFKFB4 is an independent prognostic factor in breast cancer.

Dasgupta et al. found that PFKFB4 can phosphorylate steroid receptor coactivator-3 (SRC3) and lead to increased ER co-activation and cell proliferation. The authors examined 80 samples from the Cancer Genome Atlas and demonstrated that breast cancer patients with high SRC3 and *PFKFB4* mRNA expression have unfavorable prognosis [6]. Using public high-throughput expression data, Ros et al. reported that a high level of *PFKFB4* mRNA predicted reduced survival in patients with breast cancer and non-small cell lung cancer [15]. *PFKFB4* mRNA expression has been proven to be a prognostic marker in non-muscle-invasive bladder cancer [16]. However, quantification of mRNA expression is not easy to perform in routine clinical settings. In this study, we confirmed the prognostic value of PFKFB4 protein in breast cancer using immunochemistry, which can be easily performed in FFPE samples. To the best of our knowledge, this is the first study supporting the prognostic value of PFKFB4 protein in breast cancer.

PFKFB4 plays an important role in regulating glucose metabolism and directing metabolic pathways required for biosynthesis of macromolecules to maintain cancer cell proliferation [17]. Several groups independently identified PFKFB4 as a key metabolic enzyme in cancer using high-content screening [6–8]. PFKFB4 is required to maintain the balance of glycolytic activity for energy generation and cellular redox in prostate cancer [7]. Using an unbiased RNA interference genome-wide screening assay, Dasgupta et al. discovered PFKFB4 as a dominant modulator of SRC3-dependent cancer cell proliferation [6]. PFKFB4 and SRC-3, an ER co-activator, can hyperactivate ER activity in the presence of estradiol [6], which may explain the correlation between reduced DFS and high PFKFB4 observed in luminal and ER-positive breast cancer. PFKFB4 and SRC-3 are drivers of the growth of basal-subtype breast cancer [6]. This may partially explain the prognostic significance of PFKFB4 in triple-negative and ER-negative subgroups. Further study is needed to determine the expression pattern of PFKFB4 and SRC-3 and the activated status of the PFKFB4-SRC-3 axis in breast cancer. Besides, it is also worthy to note the non-metabolic function of PFKFB4 that are relevant in cancer development. Gao et al. reported that PFKFB4 enhances breast cancer migration by induction of hyaluronan production in a p38-dependent manner [18]. Moreover, PFKFB4 can interact with endothelial tyrosine kinase to modulate chemoresistance of small-cell lung cancer by regulating autophagy [19].

Recent studies reported PFKFB4 as a potential target in cancer. Silencing of PFKFB4 induced apoptosis in p53-deficient cancer cells and inhibited tumor growth [15]. A selective PFKFB4 inhibitor, 5-(*n*-(8-methoxy-4-quinolyl)amino)pentyl nitrate, suppressed the glycolysis process and proliferation in human cancer cell lines rather non-transformed epithelial cells in vitro, suggesting that targeting PFKFB4 may be a promising therapeutic strategy against breast cancer. Our study revealed that almost half (49.0%, 98/200) of the breast cancer cases in our study had a score 3 (the highest) for PFKFB4 staining, which indicate a large population of breast cancer patients deposit the potential therapeutic target.

This study has some limitations. First, this is a retrospective study, in which we tested the association between PFKFB4 expression with DFS and OS in breast cancer rather than true prediction. Additional study is needed to validate the prognostic value of the novel marker. Second, the number of stage III patients with breast cancer in our cohort was too small (*n* = 8). Thus, the effectiveness of this potential prognostic marker should be applied to this subgroup with caution. Third, even though the study included subjects with all subtypes, the composition of the cohort was not representative of the general patient population in the real world. Larger cohorts are therefore required before PFKFB4 can be recommended for clinical practice.

## Conclusion

Our study indicates the prognostic value of PFKFB4 protein in breast cancer. High expression of PFKFB4 was associated with reduced DFS and OS in breast cancer. Our findings may therefore promote the further clinical use of PFKFB4 and provides additional prognostic information to oncologists with regard to evaluating risk via assessing cancer metabolic enzymes. Our results, together with previous mechanistic studies, provide a rationale for further clinical investigation to treat cancer by manipulating PFKFB4 expression.

## Additional file

**Additional file 1: Figure S1.** Kaplan–Meier analysis of PFKFB4 in breast cancer stratified by ER status. (**A**) DFS in ER-positive patients (*n* = 88); (**B**) OS in ER-positive patients (*n* = 88); (**C**) DFS in ER-negative patients (*n* = 112); (**D**) OS in ER-negative patient (*n* = 112). DFS, disease-free survival; OS, overall survival; ER, estrogen receptor.

## Data Availability

Not applicable.
