# Supplementary figures and images for: High expression of metabolic enzyme PFKFB4 is associated with poor prognosis of operable breast cancer
Source: Cancer Cell Int. 2019 Jun 18;19:165. doi: 10.1186/s12935-019-0882-2 (PMC6582605; doi:10.1186/s12935-019-0882-2)

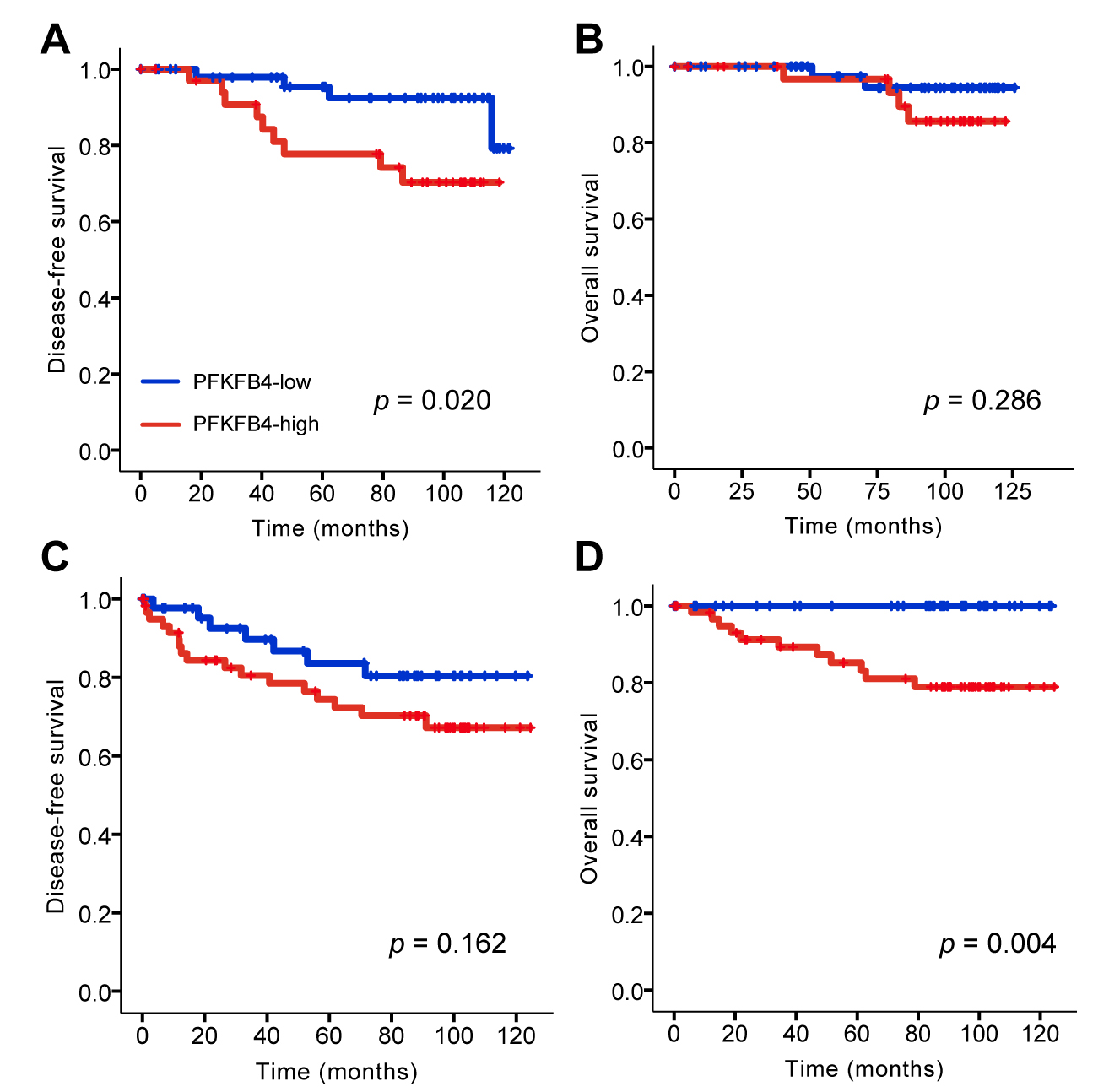

Supplement: Supplementary file 1 — Additional file 1: Figure S1. Kaplan–Meier analysis of PFKFB4 in breast cancer stratified by ER status. (A) DFS in ER-positive patients (n = 88); (B) OS in ER-positive patients (n = 88); (C) DFS in ER-negative patients (n = 112); (D) OS in ER-negative patient (n = 112). DFS, disease-free survival; OS, overall survival; ER, estrogen receptor. [file 12935_2019_882_MOESM1_ESM.jpg]
